# Nutritional Inequality and Co-Morbid Chronic Disease Among Breast Cancer Survivors in China

**DOI:** 10.3390/nu16234031

**Published:** 2024-11-25

**Authors:** Juan Xia, Jinming Yu, Lindi Qu, Lin Lv, Wenyu Zhu, Xinyu Meng, Jian Shao, Yanxia Luo

**Affiliations:** 1 Department of Epidemiology and Health Statistics, School of Public Health, Capital Medical University, Beijing 100069, China; xiajuan@ccmu.edu.cn; 2 Department of Preventive Medicine and Health Education, School of Public Health, Fudan University, Shanghai 200032, China; jmy@fudan.edu.cn (J.Y.); 23111020035@m.fudan.edu.cn (L.L.); 22211020231@m.fudan.edu.cn (W.Z.); 22211020059@m.fudan.edu.cn (X.M.); 3 Shanghai Municipal Hospital of Traditional Chinese Medicine, Shanghai University of Traditional Chinese Medicine, Shanghai 200071, China; ldqu0909@163.com; 4 Department of Clinical Nutrition, Northern Jiangsu People’s Hospital, Yangzhou 225001, China; shaojian2014@126.com

**Keywords:** nutrition literacy, dietary quality, comorbidities, breast cancer survivors, China

## Abstract

Background/Objectives: Breast cancer survivors often face an elevated risk of developing co-morbid chronic diseases, which may be exacerbated by nutritional inequalities. This study aimed to comprehensively assess the associations between nutrition literacy, dietary quality, and the risk of co-morbidity in breast cancer survivors in China. Methods: A cross-sectional study was conducted among cancer survivors enrolled in the Shanghai Cancer Rehabilitation Club from March to July 2023. The multivariable models of logistic regression, Poisson regression, and mediation analysis were used to explore the relationship between nutrition literacy, dietary quality, and co-morbid chronic diseases among breast cancer survivors in China. Results: The mean age of the 1552 female breast cancer survivors was 64.5 ± 7.02 years. Nutrition literacy was found to be low, with 49.81% of participants scoring above the threshold for adequate nutrition literacy. The median (IQR) diet quality distance, low bound score, and high bound score were 67.0 (55.0, 81.0), −39.0 (−51.0, −28.0), and 25.0 (16.0, 36.0), respectively. Of the total female breast cancer survivors, 67.27% were reported to have at least one comorbid chronic disease. Hypertension (37.32%) was the most common co-morbid chronic disease. Each score increase of 10 in nutrition literacy (AOR 0.88, 95%CI 0.808–0.962) was associated with a significant reduction in co-morbid chronic disease risk among breast cancer survivors. Poisson regression analysis of the number of chronic diseases was conducted, and consistently, an association between higher nutrition literacy levels and fewer chronic diseases was observed (AOR 0.94, 95%CI 0.911–0.971). The indirect effect of nutrition literacy on comorbidity risk through dietary quality was not significant (indirect effect = 0.994, 95%CI = 0.980–1.008). Conclusions: Nutrition literacy is paramount in breast cancer survivors’ dietary quality and prognosis. Enhancing nutrition literacy may be a crucial strategy for improving dietary quality and mitigating the risk of comorbid chronic diseases in this vulnerable population.

## 1. Introduction

Breast cancer is one of the most commonly diagnosed cancer types in the world [1] and also the second most common cancer type among women in China [2]. People with breast cancer are living longer as a result of improvements in screening and treatment [3]; the 5-year survival rates in high-income countries exceed 90% [4]. According to the latest statistics from the 2021 Global Burden of Disease study, there were nearly 4 million people living with a history of breast cancer in China [5]. Unfortunately, breast cancer survivors may experience an elevated risk of cardiometabolic diseases [6] in addition to significant aftereffects from cancer diagnoses and therapies [7]. Cardiovascular disease has been reported to be the predominant cause of death in cancer survivors other than their primary neoplasms [8,9,10], which is an emerging challenge for cardiovascular physicians and oncologists.

The World Health Organization’s Global Breast Cancer Initiative (GBCI) advocates to reduce global breast cancer mortality by 2.5% per year, thereby averting 2.5 million breast cancer deaths globally between 2020 and 2040 [4]. To achieve the goal of reducing the burden of cancer diseases and effectively safeguarding the health of broad masses of people, 13 departments, including the National Health Commission, have jointly formulated the “Healthy China Initiative-Implementation Plan for Cancer Prevention and Control (2023–2030)”. It is imperative to provide proper guidance for patients’ rehabilitation, pain management, long-term care, nutrition, and psychological support [11]. Although symptoms related to cancer therapies may persist lifelong and limit their quality of life, comorbidities are preventable by adjusting modifiable risk factors such as diet, physical activities, health behavior, etc.

Nutritional factors may play a pivotal role not only in cancer occurrence but also in mitigating comorbidities and enhancing quality of life [12,13,14]. Even though growing interest in dietary risk factors for cancer has led to numerous investigations into the association between dietary habits and breast cancer [15,16,17], the dietary landscape and its impact on the prognosis of long-term breast cancer survivors remain understudied. Considering the dramatically increased number of breast cancer survivors and the increased comorbidity burden of long-term breast cancer survivors, this study aimed to assess the nutrition status and risk of co-morbid chronic diseases among breast cancer survivors in China. Furthermore, we explored the potential impact of nutrition literacy on dietary quality and comorbidities. By identifying these associations, we hope to inform interventions that can address nutritional disparities and improve health outcomes in this vulnerable group.

## 2. Materials and Methods

This cross-sectional study was approved by the Institutional Review Board of the Capital Medical University (Z2022SY002), and all participants provided written informed consent before the survey. Reporting followed the Strengthening the Reporting of Observational Studies in Epidemiology (STROBE) reporting guideline.

### 2.1. Study Design and Participants

In this cross-sectional study, participants were recruited from the Shanghai Cancer Rehabilitation Club [18], which is a non-governmental self-help organization for people living with cancer. Now, it has more than 16,000 members, 18 branches, and 182 community blocks, forming a three-level management network of city, district, and street. The Shanghai Chancer Rehabilitation Club, established in 1989, has established 13 rehabilitation guidance centers according to the types of diseases and has also established cancer patient resource centers in 7 municipal Grade A tertiary hospitals and 1 private hospital.

Cancer survivors were enrolled and completed the questionnaires between March and July 2023. Answers to all questionnaires were collected through face-to-face interviews or through self-administered questionnaires for literate participants. This study protocol was approved by the Institutional Review Board of the Capital Medical University (Z2022SY002). Signed informed consent was obtained from all participants prior to the survey. For the purpose of this study, inclusion criteria were a history of breast cancer, completion of primary treatment (surgery, chemotherapy, radiation, and/or hormone therapy), and age 18 years or older.

### 2.2. Dietary Quality

Participants completed an interview that included a semi-quantitative food frequency questionnaire, which we used to evaluate their long-term food intake and dietary quality (scored by referring to the Diet Balance Index [19] and Healthy Eating Index [20]). We calculated the diet quality distance (DQD), low bound score (LBS), and high bound score (HBS) to evaluate the dietary quality of breast cancer survivors. When the indicator reaches the recommended amount specified in the guidelines, it is assigned a score of 0. For foods emphasized as “eat more” in the guidelines, the focus is on assessing the degree of inadequate intake; these indicators are assigned negative scores, with each decrement of “recommended amount/10” corresponding to “−1 point”. Conversely, for foods recommended to be “eaten less”, the emphasis is on evaluating the extent of excessive intake; these indicators are assigned positive values, with each increment of “recommended amount/10” corresponding to “1 point”. For foods advised to be consumed in “moderation”, the evaluation considers both inadequate and excessive intake, resulting in indicators with both positive and negative values. The values for each indicator range from −10 to 10. Additionally, a cap is imposed on each indicator’s score, with any score exceeding 10 (for positive scores) or falling below −10 (for negative scores) being adjusted to 10 or −10, respectively.

The score range for DQD is 0–160 points, based on the intake of grains, coarse grains, vegetables, dark-colored vegetables, fruits, meat and poultry, fish and shellfish, eggs, soybean and its products, dairy products, drinking water, fried foods, pickled foods, cooking oil, salt, and sugar. HBS is based on the intake of grains, meat and poultry, eggs, fried foods, pickled foods, cooking oil, salt, and sugar, ranging from 0 to 80 points. The range for LBS is 0–120 points, based on the intake of grains, coarse grains, vegetables, dark-colored vegetables, fruits, meat and poultry, fish and shellfish, eggs, soybean and its products, dairy products, drinking water, and salt. Higher diet scores indicate poorer diet quality. The ideal diet has a score of 0. The diet scores less than 20% of the total scores were defined as acceptable, 20~39.9% as mild, 40~59.9% as moderate, and higher than 60% as severe.

### 2.3. Nutrition Literacy

Nutrition literacy was measured using a validated scale, which is described in detail elsewhere [21]. It includes four dimensions, namely functional nutrition literacy, ability to obtain nutrition information, application skill, and calculation skill.

### 2.4. Primary Outcomes

The primary outcome was the status of co-morbidity (diagnosed by a medical professional) of breast cancer survivors, including hypertension, diabetes, hyperlipidemia, hyperuricemia, cardiopathy, stroke, respiratory diseases, gastrointestinal disease, and musculoskeletal disorders. We also measured the degree of multimorbidity as a count of long-term conditions.

### 2.5. Statistical Analysis

Descriptive statistics were used to summarize the participants’ characteristics. Chi-square tests and Wilcoxon rank-sum tests were performed to examine associations for categorical variables and non-normal continuous variables, respectively. We used binary logistic regression to estimate the association between nutrition literacy and co-morbid chronic disease, Poisson regression to estimate the number of co-morbid chronic diseases, and mediation analysis to estimate associations between nutrition literacy, dietary quality, and co-morbid chronic disease among breast cancer survivors. All multivariable models adjusted for age, education level, body mass index (BMI, calculated from height and weight), living situation, personal income, and physical activity. The effects and percentile-based confidence intervals of the Poisson regression were based on 5000 bootstrap samples. All statistical analyses were performed using SAS version 9.4 (SAS Institute Inc., Cary, NC, USA), and visualization of the results was implemented in GraphPad Prism version 9 (https://www.graphpad.com/ (accessed on 3 September 2024)). A 2-sided *p* value of <0.05 was considered statistically significant.

## 3. Results

### 3.1. Characteristics of the Participants

The demographic characteristics of breast cancer survivors, according to nutrition literacy and its different dimensions, are shown in Table 1. Of the total 1552 breast cancer survivors, the mean age was 64.5 ± 7.02 years; 49.74% had an adequate nutrition literacy. As for the different dimensions, 49.16% of the breast cancer survivors had adequate functional nutrition literacy, 48.07% had the ability to acquire nutrition information, 51.61% had the skills of applying nutrition knowledge, and 34.09% had the skills of calculation. The proportion of breast cancer survivors with adequate nutrition literacy is presented to increase with the levels of education and personal income (*p* < 0.001). No statistically significant difference was found between nutrition literacy and the factors of gender, age, and living situation (*p* > 0.05). Breast cancer survivors who engaged in less physical activity, who had a higher BMI, and who had comorbid chronic conditions were significantly less likely to have adequate nutrition literacy.

### 3.2. Nutrition Literacy and Comorbid Chronic Conditions

**Figure 1:**
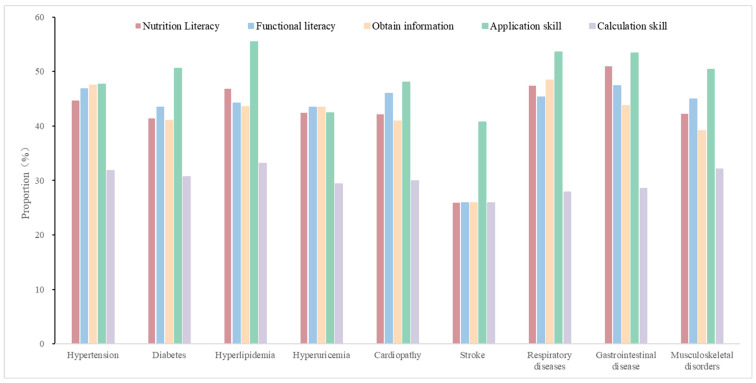
Nutrition literacy in cancer survivors with different types of co-morbid chronic diseases.

The breast cancer survivors combined with stroke had the lowest level of nutrition literacy, which was only 25.93% with adequate nutrition literacy. In contrast, those combined with gastrointestinal disease reported a higher level of nutrition literacy (51.0%). As for the different dimensions, it was similar to nutrition literacy in that breast cancer survivors combined with stroke also had the lowest level in the four dimensions (Figure 1). For the dimension of functional nutrition literacy, it was also highest in those combined with gastrointestinal disease (47.39%). However, for the ability to obtain information, it was highest in breast cancer survivors combined with respiratory diseases (48.45%); those combined with hyperlipidemia reported the highest skills of applying nutrition knowledge (55.52%) and calculation (33.14%).

### 3.3. Food Intake and Diet Quality According to Nutrition Literacy Levels

Breast cancer survivors with higher nutrition literacy had better dietary practices. Compared with breast cancer survivors who had adequate nutrition literacy, those with inadequate nutrition literacy had a significantly lower intake of wheat and products, coarse grains, poultry, eggs, dark-colored vegetables, fruits, nuts, and dairy products, while the intake of pickled foods and cooking oil was higher. The median (interquartile range; IQR) score of diet quality distance was 71.0 (58.0–85.0) for breast cancer survivors with inadequate nutrition literacy, which was significantly higher than for those with adequate nutrition literacy (63.5 [52.0–77.0]). This significant difference also appeared in the low bound score, which indicates insufficient intake; however, there was no statistical difference in the high bound score, which indicates excessive intake (Table 2).

### 3.4. Dietary Quality and Comorbid Chronic Disease

**Figure 2:**
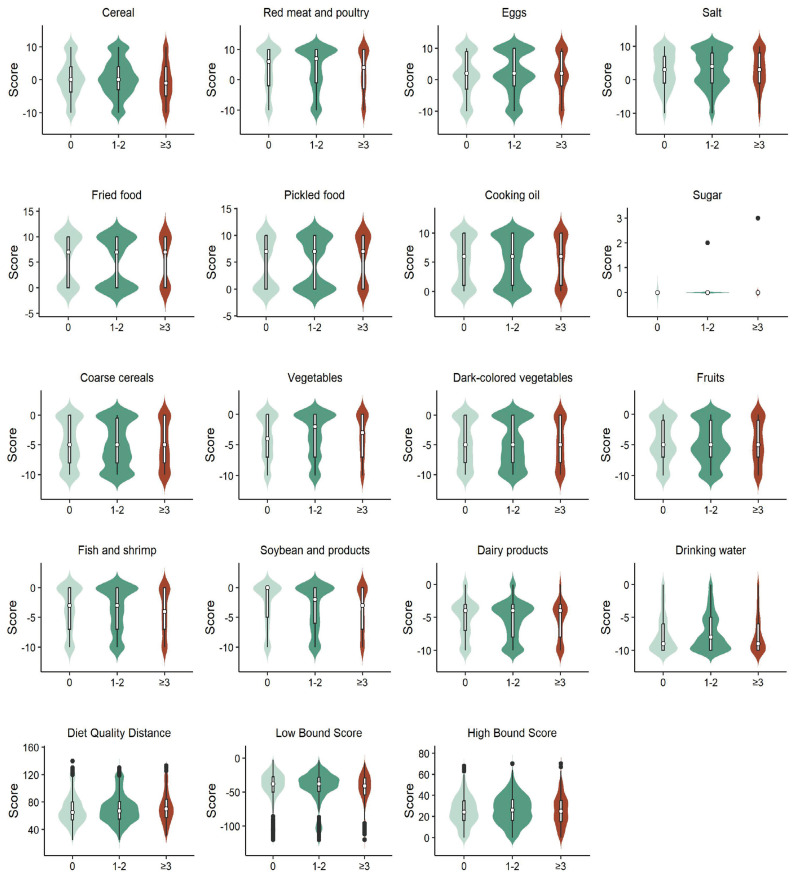
Dietary status of breast cancer survivors with different numbers of chronic diseases.

The mean diet quality distance, low bound score, and high bound score were 67.0 (55.0, 81.0), −39.0 (−51.0, −28.0), and 25.0 (16.0, 36.0), respectively. The estimated scores of dietary components for breast cancer survivors with chronic diseases differed significantly from those for breast cancer survivors without chronic disease, except for cereal and sugar. The main dietary problems of cancer survivors were excessive intake of red meat, fried foods, and pickled foods and insufficient intake of coarse grains, dark-colored vegetables, fruits, and drinking water (Figure 2).

### 3.5. Nutrition Literacy and Co-Morbid Chronic Disease

In the multivariate-adjusted analysis of this present study, each score increase of 10 in nutrition literacy (AOR 0.88, 95%CI 0.808–0.962), the dimension of functional literacy (AOR 0.88, 95%CI 0.791–0.978), and the dimension of ability to obtain information (AOR 0.77, 95%CI 0.614–0.957) were associated with a significant reduction in CVD risk among breast cancer survivors. Poisson regression analysis of the number of chronic diseases was also conducted, and consistently, an association between higher nutrition literacy level and fewer chronic diseases was observed. There was no significant difference in dimension between application skill and calculation skill (Table 3).

**Figure 3:**
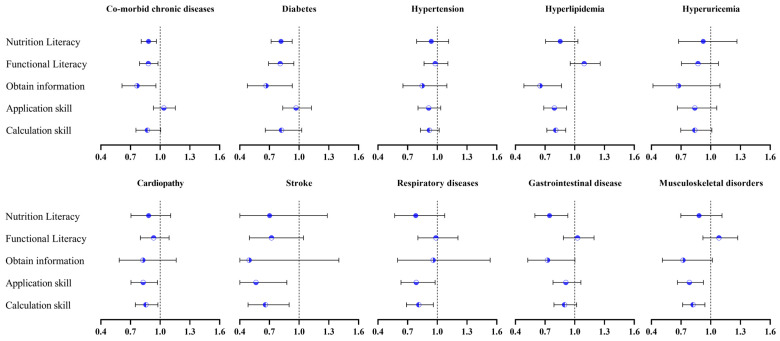
Effect estimates of logistic regression models for association of nutrition literacy with risk of co-morbid chronic diseases among breast cancer survivors.

As for the different kinds of co-morbid chronic diseases, a higher level of nutrition literacy was associated with a lower risk of diabetes (AOR 0.82, 95%CI 0.717–0.930), hyperlipidemia (AOR 0.81, 95%CI 0.718–0.909), cardiopathy (AOR 0.86, 95%CI 0.749–0.977), stroke (AOR 0.66, 95%CI 0.484–0.899), respiratory diseases (AOR 0.81, 95%CI 0.688–0.960), and musculoskeletal disorders (AOR 0.82, 95%CI 0.716–0.941). However, significant associations between hypertension (AOR 0.92, 95%CI 0.830–1.019), hyperuricemia (AOR 0.84, 95%CI 0.697–1.011), or gastrointestinal disease (AOR 0.90, 95%CI 0.790–1.019) and nutrition literacy were not observed (Figure 3, Tables S1–S9 in Supplementary Materials).

### 3.6. The Mediating Effect of Nutrition Literacy

The results of the mediation analysis indicated that nutrition literacy was significantly negatively associated with the risk of CVD in breast cancer survivors (AOR = 0.877, 95%CI = 0.801–0.952). The path from nutrition literacy to diet quality was also significant (β = −0.2301, *p* < 0.001); however, the path from diet quality to CVD risk was not significant (β = 0.0246, *p* = 0.4301). As shown in Table 4, the indirect effect of nutrition literacy on CVD risk through diet quality was not significant (indirect effect = 0.994, 95%CI = 0.980–1.008). The direct effect of nutrition literacy on CVD risk, controlling for diet quality, was still significant (β = −0.126, *p* < 0.001). The percentage decompositions of the total effect can be found in Table S10 in Supplementary Materials.

## 4. Discussion

In this sample of breast cancer survivors in Shanghai Cancer Rehabilitation Club, we explored nutritional inequality in the context of co-morbid chronic diseases among breast cancer survivors, with a particular focus on nutrition literacy, dietary quality, and their associations with chronic disease risk. Our findings reveal a notable nutritional inequality among breast cancer survivors, characterized by low levels of nutrition literacy and poor dietary quality. The low nutrition literacy contributed to suboptimal dietary choices and increased risk of co-morbid chronic diseases, highlighting the need for targeted interventions to address these disparities and improve health outcomes in this vulnerable group.

Consistent with previous findings [22,23], our results also indicate a significant correlation between nutrition literacy and dietary behaviors. A generally low level of nutrition literacy and poor dietary quality among breast cancer survivors observed in our study is particularly concerning, as adequate nutrition is paramount for optimal recovery, management of treatment side effects, and reduction of recurrence and comorbidity risk [24,25,26,27]. Previous studies have confirmed that dietary factors have a significant impact on stroke risk [28,29,30], and similar results are reflected in breast cancer survivors. Our results show that breast cancer survivors with concurrent stroke generally exhibit lower levels of nutrition literacy, with only 25.93% of breast cancer survivors having adequate nutrition literacy.

The knowledge-attitude-practice (KAP) theory model serves as a widely utilized theoretical framework in health education and behavioral intervention [31], positing that knowledge forms the foundation for developing accurate attitudes and altering practices, whereas attitude acts as the impetus for practicing changes. Consistent with the KAP theory, the results of this study show that breast cancer survivors with lower levels of nutrition literacy correspond to unfavorable food intake, which leads to a state of suboptimal dietary quality. Specifically, the diet quality distance of breast cancer survivors with inadequate nutrition literacy was significantly higher than those with adequate nutrition literacy (*p* < 0.001). Low nutrition literacy may stem from inadequate access to nutritional education, misinformation, or a lack of understanding regarding the specific dietary needs of cancer survivors [22,32,33]. Addressing this gap through targeted educational interventions, such as nutrition counseling and workshops tailored to breast cancer survivors, could significantly improve their nutritional knowledge and empower them to make healthier food choices, thereby achieving the prevention of chronic diseases through optimal dietary patterns [34].

The results of this study revealed that low levels of nutrition literacy were significantly associated with a higher risk of co-morbid chronic diseases in breast cancer survivors, and this association was not mediated by diet quality. This underscores the importance of nutrition literacy as a modifiable factor that can influence health outcomes after cancer treatment. Unlike with the results of studies in non-cancer populations [35,36,37], we did not observe a statistically significant association between diet quality and the risk of comorbid chronic diseases in breast cancer survivors. In addition to the differences in population characteristics, the widespread nature of poor dietary practices among breast cancer survivors may have obscured the potential associations between dietary quality and co-morbid chronic disease outcomes. Specifically, the proportion of breast cancer survivors in this study with the dietary quality of acceptable, mild, moderate, and severe dietary imbalance was 1.49%, 43.66%, 42.56%, and 12.29, respectively, which was significantly worse than that of the general population. Nevertheless, this result highlights the need for further research to elucidate the role of dietary quality in the development of comorbid chronic diseases in this population living with cancer.

Furthermore, the findings of this study support the use of a simple and easily measurable nutrition literacy scale to predict the risk of co-morbid chronic diseases among cancer survivors. It not only reflects dietary quality [38] and provides a more sensitive prediction of co-morbid chronic disease risk but may also be more feasible to implement in large-scale epidemiological investigations than complex dietary assessments. According to the KAP theory, by enhancing nutritional literacy, breast cancer survivors may be better equipped to make informed dietary choices that support their overall health and mitigate the risk of developing chronic diseases. Given that this vulnerable population in our study lacked the necessary knowledge and skills to make informed dietary choices and manage chronic conditions effectively, future research should investigate the effectiveness of various educational strategies in improving nutritional literacy and subsequent health outcomes among breast cancer survivors.

The strengths of this study include a community-based setting, which enhances the relevance and applicability of the findings to real-world settings; a detailed collection of nutrition literacy and lifestyle, dietary, and health outcome data, allowing for adjustment for potential confounders; and comprehensive assessments of nutrition literacy, dietary quality, and their associations with co-morbid chronic diseases, which provide a more holistic understanding of the nutritional inequality faced by breast cancer survivors. By focusing on this distinctively vulnerable population, which often experiences unique challenges related to nutrition and health, the research contributes to bridging a notable gap within the existing literature and highlighting several critical points that warrant further exploration and intervention.

The study limitations should be acknowledged. Considering its cross-sectional design, causal relationships cannot be precisely inferred. While associations were observed, longitudinal data would be necessary to confirm the temporal sequence and causality of these relationships. Furthermore, due to constraints in time and manpower, this survey was only conducted among members of the Shanghai Cancer Rehabilitation Club, which may limit the generalizability of our findings to the broader population of breast cancer survivors. Given that the cancer survivors in the cancer rehabilitation club might have distinct characteristics, such as higher levels of social support or specific health-seeking behaviors, it is inferred that nutritional inequalities might be more pronounced in those cancer survivors who do not join the cancer rehabilitation club. This is the first step in a series of studies; further studies are needed to expand the survey scope and explore the nutritional inequalities associated with co-morbid chronic diseases in the future. Moreover, dietary quality relied on a self-reported food frequency questionnaire, which may be subject to recall bias, and the participants had universally poor dietary habits, which might be reasons why no statistical association between diet quality and co-morbid chronic diseases was observed. Objective biomarkers that could provide more accurate assessments of nutritional status and the inclusion of participants with a variety of dietary qualities are needed in future studies to further elucidate the complex relationships between nutritional inequality and co-morbid chronic diseases among breast cancer survivors.

## 5. Conclusions

In conclusion, our study highlights the prevalence of low nutrition literacy and poor dietary quality among breast cancer survivors, as well as the significant association between low nutrition literacy and increased risk of co-morbid chronic diseases. These findings underscore the urgent need for targeted nutritional education and interventions to improve the health outcomes of this vulnerable population. Improving nutrition literacy and dietary quality for cancer survivors should be an integral part of their post-treatment care plans, and underscoring the crucial role that nutrition plays in optimizing their prognosis is of great significance in reducing the disease burden of cancer in our country. Future research should focus on identifying effective strategies to enhance nutritional literacy and exploring the potential biological mechanisms of diet in the prevention and management of comorbid chronic diseases in breast cancer survivors. Ultimately, addressing nutritional inequality among breast cancer survivors requires a multifaceted approach that integrates education, policy, and healthcare services to ensure optimal nutrition and overall well-being.

## Figures and Tables

**Table 1:** Characteristics of the participants by nutrition literacy [n (%)].

Characteristics	Functional Nutrition Literacy	Information	Application	Calculation	Nutrition Literacy
Sex					
Male	3 (50.00)	2 (33.33)	3 (50.00)	1 (16.67)	2 (33.33)
Female	760 (49.16)	744 (48.12)	798 (51.62)	528 (34.15)	770 (49.81)
$\chi^{2}$	0.002	0.524	0.006	0.813	0.649
*p*-value	0.967	0.469	0.937	0.367	0.421
Age group					
<60	200 (49.63)	234 (58.06)	232 (57.57)	162 (40.20)	220 (54.59)
60~65	162 (48.36)	150 (44.78)	159 (47.46)	120 (35.82)	166 (49.55)
65~69	230 (49.68)	214 (46.22)	226 (48.81)	154 (33.26)	218 (47.08)
≥70	165 (50.00)	136 (41.21)	177 (53.64)	91 (27.58)	161 (48.79)
$\chi^{2}$	0.214	24.429	9.994	13.376	5.192
*p*-value	0.975	<0.001	0.019	0.004	0.158
Education					
Primary and below	40 (39.22)	32 (31.37)	30 (29.41)	22 (21.57)	34 (33.33)
Junior high school	289 (43.99)	307 (46.73)	282 (42.92)	201 (30.59)	292 (44.44)
Senior high school	283 (54.21)	267 (51.15)	324 (62.07)	192 (36.78)	291 (55.75)
College or above	96 (64.86)	93 (62.84)	118 (79.73)	78 (52.70)	105 (70.95)
$\chi^{2}$	30.919	26.344	109.163	34.888	52.162
*p*-value	<0.001	<0.001	<0.001	<0.001	<0.001
Inhabiting information					
With spouse	350 (51.17)	322 (47.08)	342 (50.00)	236 (34.50)	342 (50.00)
With family	57 (50.00)	53 (46.49)	64 (56.14)	43 (37.72)	62 (54.39)
With spouse and family	275 (46.69)	292 (49.58)	304 (51.61)	201 (34.13)	289 (49.07)
Live alone	80 (50.00)	77 (48.13)	90 (56.25)	48 (30.00)	79 (49.38)
$\chi^{2}$	2.615	0.919	3.020	1.910	1.102
*p*-value	0.455	0.821	0.389	0.591	0.777
Self income (¥)					
≤1000	52 (41.94)	44 (35.48)	42 (33.87)	30 (24.19)	47 (37.90)
1001~3000	198 (43.61)	217 (47.80)	194 (42.73)	139 (30.62)	200 (44.05)
3001~5000	389 (50.32)	366 (47.35)	442 (57.18)	276 (35.71)	396 (51.23)
5001~8000	96 (63.58)	90 (59.60)	99 (65.56)	64 (42.38)	100 (66.23)
>8000	21 (61.76)	23 (67.65)	18 (52.94)	13 (38.24)	23 (67.65)
$\chi^{2}$	23.313	21.299	51.349	13.636	34.274
*p*-value	<0.001	<0.001	<0.001	0.009	<0.001
Physical activity (times per week)					
5~7	228 (57.87)	205 (52.03)	203 (51.52)	137 (34.77)	225 (57.11)
3~4	274 (53.52)	259 (50.59)	307 (59.96)	198 (38.67)	288 (56.25)
1~2	162 (43.32)	175 (46.79)	167 (44.65)	124 (33.16)	159 (42.51)
<1	79 (36.74)	83 (38.60)	105 (48.84)	57 (26.51)	80 (37.21)
$\chi^{2}$	34.031	11.701	21.942	10.325	38.361
*p*-value	<0.001	0.009	<0.001	0.016	<0.001
BMI					
Underweight	22 (56.41)	14 (35.90)	26 (66.67)	18 (46.15)	22 (56.41)
Normal	417 (50.61)	396 (48.06)	457 (55.46)	297 (36.04)	431 (52.31)
Overweight	265 (49.53)	260 (48.60)	261 (48.79)	170 (31.78)	259 (48.41)
Obesity	52 (36.62)	73 (51.41)	51 (35.92)	38 (26.76)	54 (38.03)
$\chi^{2}$	10.473	2.989	24.149	8.601	11.033
*p*-value	0.015	0.393	<0.001	0.035	0.012
Number of chronic diseases					
0	272 (53.65)	276 (54.44)	265 (52.27)	200 (39.45)	283 (55.82)
1–2	368 (48.74)	345 (45.70)	397 (52.58)	244 (32.32)	367 (48.61)
≥3	123 (42.41)	125 (43.10)	139 (47.93)	85 (29.31)	122 (42.07)
$\chi^{2}$	9.421	12.807	1.946	10.482	14.706
*p*-value	0.009	0.002	0.378	0.005	0.001

Abbreviations: BMI, body mass index.

**Table 2:** Food intake and diet quality in breast cancer survivors (median [IQR]).

	Total	Inadequate Nutrition Literacy	Adequate Nutrition Literacy	*p* Value
Rice and products	171.4 (100.0, 214.3)	200.0 (100.0, 257.1)	171.4 (100.0, 214.3)	0.051
Wheat and products	50.0 (28.6, 100.0)	42.9 (28.6, 85.7)	57.1 (28.6, 100.0)	<0.001
Coarse grains	42.9 (14.3, 75.0)	28.6 (14.3, 71.4)	42.9 (21.4, 85.7)	0.039
Fried foods	7.1 (0.0, 14.3)	7.1 (0.0, 14.3)	7.1 (0.0, 14.3)	0.072
Pork	42.9 (28.6, 85.7)	42.9 (28.6, 85.7)	42.9 (28.6, 71.4)	0.505
Beef and lamb	14.3 (7.1, 28.6)	14.3 (7.1, 28.6)	14.3 (7.1, 28.6)	0.167
Poultry	14.3 (14.3, 42.9)	14.3 (14.3, 28.6)	15.7 (14.3, 42.9)	0.041
Eggs	42.9 (21.4, 71.4)	42.9 (21.4, 71.4)	42.9 (28.6, 71.4)	0.014
Fish and shellfish	50.0 (42.9, 100.0)	50.0 (42.9, 100.0)	50.0 (50.0, 100.0)	0.231
Vegetables	300.0 (200.0, 500.0)	300.0 (160.7, 500.0)	300.0 (200.0, 500.0)	0.800
Dark-colored vegetables	100.0 (57.1, 200.0)	100.0 (42.9, 200.0)	114.3 (64.3, 250.0)	<0.001
Soybean and products	35.7 (14.3, 71.4)	28.6 (14.3, 71.4)	35.7 (21.4, 71.4)	0.107
Pickled food	7.1 (0.0, 14.3)	7.1 (0.0, 14.3)	7.1 (0.0, 14.3)	0.004
Fruits	142.9 (100.0, 214.3)	128.6 (85.7, 200.0)	150.0 (100.0, 250.0)	0.009
Nuts	28.6 (14.3, 50.0)	28.6 (14.3, 50.0)	28.6 (14.3, 50.0)	0.032
Dairy products	250.0 (142.9, 250.0)	250.0 (107.1, 250.0)	250.0 (142.9, 250.0)	0.022
Cooking oil	40.0 (27.8, 55.6)	40.0 (27.8, 55.6)	39.2 (27.8, 50.0)	0.006
Salt	5.0 (2.9, 5.8)	5.0 (2.9, 5.8)	4.7 (2.9, 5.8)	0.262
Sugar	2.8 (0.5, 5.6)	2.8 (0.5, 5.6)	2.8 (0.6, 5.6)	0.254
Drinking water	1000.0 (800.0, 1500.0)	1000.0 (600.0, 1500.0)	1000.0 (800.0, 1500.0)	<0.001
Diet quality distance	67.0 (55.0, 81.0)	71.0 (58.0, 85.0)	63.5 (52.0, 77.0)	<0.001
Low bound score	−39.0 (−51.0, −28.0)	−41.0 (−55.0, −30.0)	−36.0 (−48.0, −25.0)	<0.001
High bound score	25.0 (16.0, 36.0)	26.0 (16.0, 37.0)	25.0 (16.0, 35.0)	0.162

Abbreviations: IQR, interquartile range.

**Table 3:** Summary results of multivariable models of co-morbid chronic disease risk among breast cancer survivors.

Variable	Logistic		Poisson	
	AOR (95%CI)	*p* Value	AOR (95%CI)	*p* Value
Nutrition literacy	0.882 (0.808–0.962)	0.005	0.940 (0.911–0.971)	0.0002
Functional literacy	0.880 (0.791–0.978)	0.018	0.929 (0.893–0.967)	0.0003
Obtain information	0.766 (0.614–0.957)	0.019	0.908 (0.835–0.987)	0.0239
Application skill	1.037 (0.932–1.153)	0.505	1.000 (0.962–1.041)	0.9846
Calculation skill	0.871 (0.755–1.005)	0.059	0.961 (0.907–1.019)	0.1838

**Table 4:** Estimation of mediating effects adjusting for confounding covariates.

	Estimate	Standard Error	95%CI	*p* Value
Total Effect	0.877	0.039	0.801–0.952	0.001
Controlled direct effect (CDE)	0.882	0.039	0.805–0.959	0.003
Natural direct effect (NDE)	0.882	0.039	0.805–0.959	0.003
Natural indirect effect (NIE)	0.994	0.007	0.980–1.008	0.433
Percentage mediated	4.027	5.351	−6.460–14.514	0.452
Percentage due to interaction	−0.157	0.142	−0.434–0.120	0.268
Percentage eliminated	4.411	5.939	−7.230–16.052	0.458

## Data Availability

The datasets used in the current study are available on reasonable request with the permission of the corresponding author.

## Supplementary Materials

The following supporting information can be downloaded at: https://www.mdpi.com/article/10.3390/nu16234031/s1, Table S1: Effect estimates of logistic regression models for association of nutrition literacy with diabetes among breast cancer survivors; Table S2: Effect estimates of logistic regression models for association of nutrition literacy with hypertension among breast cancer survivors; Table S3: Effect estimates of logistic regression models for association of nutrition literacy with hyperlipidemia among breast cancer survivors; Table S4: Effect estimates of logistic regression models for association of nutrition literacy with hyperuricemia among breast cancer survivors; Table S5: Effect estimates of logistic regression models for association of nutrition literacy with cardiopathy among breast cancer survivors; Table S6: Effect estimates of logistic regression models for association of nutrition literacy with stroke among breast cancer survivors; Table S7: Effect estimates of logistic regression models for association of nutrition literacy with respiratory diseases among breast cancer survivors; Table S8: Effect estimates of logistic regression models for association of nutrition literacy with gastrointestinal disease among breast cancer survivors; Table S9: Effect estimates of logistic regression models for association of nutrition literacy with musculoskeletal disorders among breast cancer survivors; Table S10: Percentage decompositions of the total effect.
