# Supplementary material for: Nutritional Inequality and Co-Morbid Chronic Disease Among Breast Cancer Survivors in China
Source: Nutrients. 2024 Nov 25;16(23):4031. doi: 10.3390/nu16234031 (PMC11643076; doi:10.3390/nu16234031)
Supplement: Supplementary file 1 [file nutrients-16-04031-s001.zip › nutrients-3292656-supplementary.pdf]

Supplementary Materials

Table S1. Effect estimates of logistic regression models for association of nutrition literacy with diabetes among breast cancer survivors

Variable	Unadjusted		Adjusted	
	OR (95%CI)	*P* value	OR (95%CI)	*P* value
Nutrition literacy	0.811 (0.732-0.899)	<0.001	0.817 (0.717-0.930)	0.002
Functional literacy	0.818 (0.721-0.929)	0.002	0.809 (0.691-0.947)	0.008
Obtain information	0.587 (0.448-0.768)	<0.001	0.666 (0.477-0.931)	0.017
Application skill	0.971 (0.863-1.093)	0.624	0.969 (0.835-1.125)	0.678
Calculation skill	0.800 (0.662-0.966)	0.021	0.822 (0.658-1.027)	0.085

Table S2. Effect estimates of logistic regression models for association of nutrition literacy with hypertension among breast cancer survivors

Variable	Unadjusted		Adjusted	
	OR (95%CI)	*P* value	OR (95%CI)	*P* value
Nutrition literacy	0.859 (0.793-0.931)	<0.001	0.920 (0.830-1.019)	0.110
Functional literacy	0.857 (0.776-0.947)	0.002	0.913 (0.805-1.035)	0.153
Obtain information	0.735 (0.597-0.906)	0.004	0.847 (0.653-1.097)	0.208
Application skill	0.926 (0.843-1.017)	0.109	0.979 (0.866-1.107)	0.738
Calculation skill	0.828 (0.718-0.955)	0.010	0.939 (0.791-1.115)	0.473

Table S3. Effect estimates of logistic regression models for association of nutrition literacy with hyperlipidemia among breast cancer survivors

Variable	Unadjusted		Adjusted	
	OR (95%CI)	*P* value	OR (95%CI)	*P* value
Nutrition literacy	0.835 (0.761-0.915)	<0.001	0.808 (0.718-0.909)	<0.001
Functional literacy	0.835 (0.743-0.939)	0.003	0.794 (0.687-0.917)	0.002
Obtain information	0.627 (0.493-0.798)	<0.001	0.649 (0.486-0.866)	0.003
Application skill	1.039 (0.933-1.158)	0.484	1.095 (0.954-1.257)	0.197
Calculation skill	0.847 (0.717-1.000)	0.049	0.853 (0.705-1.032)	0.102

Table S4. Effect estimates of logistic regression models for association of nutrition literacy with hyperuricemia among breast cancer survivors

Variable	Unadjusted		Adjusted	
	OR (95%CI)	*P* value	OR (95%CI)	*P* value
Nutrition literacy	0.791 (0.683-0.915)	0.002	0.839 (0.697-1.011)	0.066
Functional literacy	0.800 (0.663-0.966)	0.020	0.839 (0.663-1.061)	0.143
Obtain information	0.572 (0.391-0.836)	0.004	0.674 (0.416-1.093)	0.110
Application skill	0.802 (0.680-0.946)	0.009	0.871 (0.704-1.078)	0.204
Calculation skill	0.809 (0.610-1.072)	0.141	0.924 (0.675-1.265)	0.622

Table S5. Effect estimates of logistic regression models for association of nutrition literacy with cardiopathy among breast cancer survivors

Variable	Unadjusted	Adjusted

	OR (95%CI)	*P* value	OR (95%CI)	*P* value
Nutrition literacy	0.801 (0.721-0.889)	<0.001	0.856 (0.749-0.977)	0.022
Functional literacy	0.815 (0.716-0.928)	0.002	0.828 (0.705-0.973)	0.022
Obtain information	0.605 (0.462-0.793)	<0.001	0.825 (0.586-1.162)	0.271
Application skill	0.881 (0.782-0.992)	0.036	0.934 (0.800-1.090)	0.385
Calculation skill	0.829 (0.686-1.001)	0.051	0.883 (0.705-1.106)	0.278

Table S6. Effect estimates of logistic regression models for association of nutrition literacy with stroke among breast cancer survivors

Variable	Unadjusted		Adjusted	
	OR (95%CI)	*P* value	OR (95%CI)	*P* value
Nutrition literacy	0.661 (0.527-0.830)	<0.001	0.660 (0.484-0.899)	0.008
Functional literacy	0.654 (0.474-0.902)	0.010	0.564 (0.364-0.876)	0.011
Obtain information	0.463 (0.240-0.893)	0.022	0.495 (0.175-1.402)	0.186
Application skill	0.807 (0.612-1.066)	0.131	0.721 (0.498-1.044)	0.083
Calculation skill	0.770 (0.462-1.281)	0.314	0.702 (0.384-1.285)	0.252

Table S7. Effect estimates of logistic regression models for association of nutrition literacy with respiratory diseases among breast cancer survivors

Variable	Unadjusted		Adjusted	
	OR (95%CI)	*P* value	OR (95%CI)	*P* value
Nutrition literacy	0.847 (0.739-0.970)	0.017	0.813 (0.688-0.960)	0.015
Functional literacy	0.859 (0.717-1.030)	0.100	0.787 (0.633-0.978)	0.031
Obtain information	0.787 (0.540-1.145)	0.210	0.958 (0.599-1.534)	0.859
Application skill	0.945 (0.801-1.115)	0.503	0.986 (0.804-1.209)	0.895
Calculation skill	0.827 (0.631-1.083)	0.167	0.782 (0.569-1.075)	0.130

Table S8. Effect estimates of logistic regression models for association of nutrition literacy with gastrointestinal disease among breast cancer survivors

Variable	Unadjusted		Adjusted	
	OR (95%CI)	*P* value	OR (95%CI)	*P* value
Nutrition literacy	0.896 (0.810-0.991)	0.033	0.897 (0.790-1.019)	0.096
Functional literacy	0.917 (0.808-1.041)	0.179	0.910 (0.780-1.062)	0.230
Obtain information	0.672 (0.514-0.877)	0.003	0.725 (0.525-1.002)	0.051
Application skill	0.999 (0.886-1.126)	0.988	1.028 (0.885-1.195)	0.716
Calculation skill	0.775 (0.640-0.939)	0.009	0.745 (0.597-0.930)	0.009

Table S9. Effect estimates of logistic regression models for association of nutrition literacy with musculoskeletal disorders among breast cancer survivors

Variable	Unadjusted		Adjusted	
	OR (95%CI)	*P* value	OR (95%CI)	*P* value
Nutrition literacy	0.802 (0.723-0.889)	<0.001	0.821 (0.716-0.941)	0.005
Functional literacy	0.796 (0.699-0.907)	0.001	0.784 (0.663-0.926)	0.004
Obtain information	0.571 (0.436-0.747)	<0.001	0.720 (0.511-1.016)	0.062

Application skill	0.964 (0.853-1.089)	0.555	1.083 (0.921-1.273)	0.336
Calculation skill	0.799 (0.658-0.971)	0.024	0.882 (0.698-1.114)	0.291

Table S10. Percentage decompositions of the total effect

Decomposition	Effect	Percent	S.E.	95% CI	*P*
NDE+NIE	Natural Direct	95.97	5.35	77.79 -107.49	<0.001
	Natural Indirect	4.03	5.35	-7.49 - 22.21	0.452
CDE+PE	Controlled Direct	95.59	5.94	76.22 -107.63	<0.001
	Portion Eliminated	4.41	5.94	-7.63 - 23.78	0.458
TDE+PIE	Total Direct	95.43	5.99	76.39 - 108.47	<0.001
	Pure Indirect	4.57	5.99	-8.47 - 23.61	0.446
NDE+PIE+IMD	Natural Direct	95.97	5.35	77.79 - 107.49	<0.001
	Pure Indirect	4.57	5.99	-8.47 - 23.61	0.446
	Mediated Interaction	-0.54	0.66	-1.81 - 0.97	0.413
CDE+PIE+PAI	Controlled Direct	95.59	5.94	76.22 - 107.63	<0.001
	Pure Indirect	4.57	5.99	-8.47 - 23.61	0.446
	Portion Due to Interaction	-0.16	0.14	-0.47 - 1.02	0.268
Four-Way	Controlled Direct	95.59	5.94	76.22 - 107.63	<0.001
	Reference Interaction	0.38	0.62	-0.28 - 2.18	0.538
	Mediated Interaction	-0.54	0.66	-1.81 - 0.97	0.413
	Pure Indirect	4.57	5.99	-8.47 - 23.61	0.446

Abbreviations: NDE, Natural direct effect; NIE, Natural indirect effect; CDE, controlled direct effect; IRF, reference interaction; IMD, mediated interaction; PIE, pure indirect effect
